# Cognitive strategies for managing cheating: The roles of cognitive abilities in managing moral shortcuts

**DOI:** 10.3758/s13423-021-01936-7

**Published:** 2021-05-19

**Authors:** Avshalom Galil, Maor Gidron, Jessica Yarmolovsky, Ronny Geva

**Affiliations:** 1 Department of Psychology, Bar Ilan University, Ramat Gan, Israel; 2 Gonda Multidisciplinary Brain Research Center, Bar Ilan University, Ramat Gan, Israel

**Keywords:** Attention, Inhibition, Eye movements and visual attention, Cognitive and attentional control

## Abstract

Cheating and immorality are highly researched phenomena, likely due to their great impact. However, little research has examined the real-time cognitive mechanisms that are involved in cheating and conflict management. Much of the cheating research to date concentrates on binary cheating; however, in more prevalent real-world scenarios, people often engage in more ambiguous self-serving mistakes. To execute such self-serving decisions, one may make use of conflict-management strategies to help balance an internal struggle between gain and self-concept. We propose that to enact such strategies one must employ sufficient cognitive resources. To test this, we employed a simple effortful control task that allows for comparisons of gain and no-gain errors, isolating self-serving mistakes while recording gaze and response-time measures. Findings revealed that individuals can make use of conflict management strategies that mimicked errors made inadvertently. Two strategies included gaze avert and quick response times during gain blocks, whereby participants simulated out-of-control-like behaviors while engaging in self-serving mistakes, plausibly as a method of self-justification. Strategy use was dependent upon individuals' cognitive abilities. Participants reporting high inhibitory control abilities were able to use gaze aversion to engage in self-serving mistakes, while those reporting high attention resources were able to employ faster response times when making more profitable errors. Taken together, this paper contributes to (1) the debate on whether honesty/dishonesty is the dominant response, (2) the debate on self-control and inhibition on cheating, and (3) the understudied area of cognitive justifications to maintain a positive self-concept.

## Introduction

Cheating and immorality are highly researched phenomena (Kochanska et al., 1997), likely due to their great impact on humans and world economies (Speights & Hilinski, 2005; Wells, 1999). However, research is needed on the real-time cognitive mechanisms involved in the conflict management and execution of such a decision. The present study aimed to examine strategies used when there is an opportunity to cheat, as well as the cognitive resources that facilitate the use of these strategies in real-time.

Much of the cheating research to date concentrates on binary cheating in which participants know that there is an explicit opportunity to cheat and they need to decide if they will go for it or not (i.e., Bucciol & Piovesan, 2011; Mazar et al., 2008). However, in more prevalent real-world scenarios, people often engage in more ambiguous rule-breaking, in which minor or seemingly insignificant self-serving mistakes are made to increase personal profit (Galil et al., 2019; Leib et al., 2019). Such self-serving mistakes may entail cutting corners or investing less effort when an error is profitable under the guise of inattention to detail or impulsive error.

Cognitive research suggests that executing such behaviors may not be easy, requiring activation of quite an extensive neuro-cognitive process. That is, as a default people tend to comply with rules; and even if rules are broken, the behavior is still attracted toward compliance (Pfister et al., 2016). Hence, executing self-serving mistakes is thought to require mental effort to adapt behavior in ways that help self-justify one's action by mimicking a mistake.

When committing a self-serving mistake one must balance the drive to increase personal gain (Hilbig & Thielmann, 2017; Kotaman, 2017; Zhao et al., 2017) while maintaining a positive self-image (Fu et al., 2016). To maintain such a balance a strategy may be used (Pittarello et al., 2015; Schweitzer & Hsee, 2002; Shalvi et al., 2011; Shalvi et al., 2012), such as investing effort in a biased way to increase the gain (Brown & Moore, 2000; Galil et al., 2019), or responding in a way that mimics a mistake. Further, the frequency with which one uses these strategies must remain low to match expected error rates. Hence, in addition to the effort needed to execute a self-serving mistake, one has to also monitor and regulate its frequency of use. This plausibly requires the investment of executive functioning, inhibition control, and attentional resources (Mazar et al., 2008).

We explored the roles of executive functions (EFs) in cheating by studying responses to high-gain opportunities as a function of inhibitory control and attention resources. Research to date dealing with populations who experience EF deficits, such as participants with attention-deficit hyperactivity disorder (ADHD), show mixed results. Some of the literature found that, compared to controls, participants with ADHD failed to truthfully report delinquent acts (Sibley et al., 2010), were more likely to be involved in unethical activities (Fletcher & Wolfe, 2009; Sibley et al., 2011), and cheated more in childhood (Hinshaw et al., 1995); while a meta-analysis found a negative association between EFs and cheating (Paulhus & Dubois, 2015). On the other hand, EFs have been found to assist in lie-telling ability (Evans & Lee, 2011, 2013; Talwar et al., 2007; Talwar & Lee, 2008) and to predict criminal activity (Babinski et al., 1999). These contradictory results suggest a more complex picture of the way EF interacts with cheating. Ding et al. (2014) shine a light on this complex interaction, showing that while children with better working memory and inhibitory control were less likely to cheat, those that did cheat showed greater cognitive flexibility and used more tactics. Other research has supported this notion, suggesting that cheating and immoral acts require cognitive resources (Dionisio et al., 2001; Gino et al., 2011) and attention allocation (Galil et al., 2019). In light of this, we suggest that cognitive abilities, namely attention resources and inhibitory control, play a role in directing strategies to cheat yet plausibly feel "OK" about it.

Research suggests that rule-based behaviors are retrieved automatically when the agent engages in other intentions (Pfister et al., 2019), and are then suppressed to enable the execution of an alternative self-centered response (Debey et al., 2014; Foerster et al., 2019). Thus plausibly, cognitive abilities are not only involved in the act of cheating, but also in enacting self-justification strategies for maintaining a positive self-image during a cheating event. One such strategy is gaze aversion. As a default, we tend to attend more to stimuli that represent the dominant desirable outcome (Halevy & Chou, 2014); therefore, gaze allocation serves a motivational role, guiding people towards information that will help them with their goals (Isaacowitz, 2006). Following this supposition, Pittarello et al. (2016) found that participants looked less at task instructions when they cheated than when they did not. This behavior suggests that gaze aversion is strategically employed during cheating, though the underlying cognitive mechanism is unclear. While gaze is considered an attentional process that enables orienting to a dominant target stimulus, gaze aversion as a strategy in cheating requires one to suppress their dominant orienting response in order to disengage from the stimulus and attend toward other non-dominant stimuli. Therefore, we propose that a strategized gaze aversion notably requires inhibitory control.

Another strategy that may be used when executing a self-serving mistake is a rapid response when a prospect for gain is known. That is, one can respond faster in a manner that resembles a decisive action or a hasty impulsive error. Response time is an important aspect of decision-making (Wise & Kong, 2005), and provides information about the amount of investment in the task. The time it takes to respond captures the participant’s cognitive ability to reach the best decision quickly while addressing all relevant considerations and regulating impulsive tendencies. Research on volitional lying and deception has suggested that acting intentionally dishonest demands a more prolonged information processing course, which is needed to consider the alternatives and suppress the dominant behavior (Foerster et al., 2019). At the same time, studies note that when a self-serving condition appears, decisions that have a high probability of being dishonest take less time and participants express less hesitation (Tabatabaeian et al., 2015). Aiming to mitigate this gap, we suggest that to employ rapid response as a strategy to increase self-serving gain yet minimize the cost to self-image, one must plan ahead and vigilantly attend to all possible motivational pulls: both the correct response as well as the high gain option. Further, this must be done rapidly to be able to complete the full cognitive and emotional process needed to be able to justify executing a self-serving mistake. Thus, we propose that in order to strategically respond faster one must have sufficient attention abilities.

**Fig. 1:**
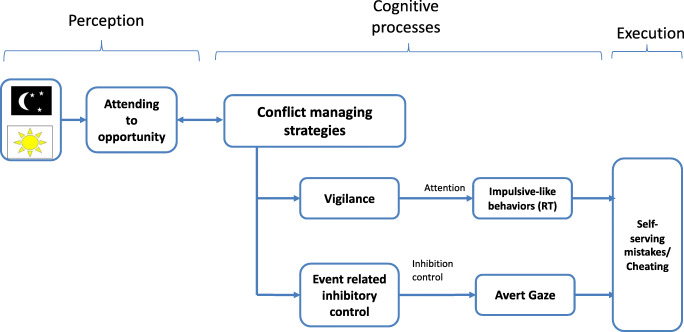
A theoretical perception and cognitive processes model, integrating cognitive mechanisms and managing-conflict strategies during cheating. The sun/moon illustration portrays an example of a choice task (e.g., a Stroop task) presented to participants. The task offers gain and no-gain opportunities allowing for differentiation between impulsive errors and self-serving mistakes that lead to personal gain

To date, research has focused on general cognitive resources and deficits that increase the probability of cheating or immoral behavior, though little is known about the interpersonal cognitive processes that aid in real-time when executing self-serving mistakes. The current study examined the specific cognitive elements that may assist in implementing cheating strategies based on cognitive inhibitory and attentional abilities. We offered participants a simple, ecologically valid task that presents opportunities to make self-serving mistakes (Galil et al., 2019), and explored the influence of self-reported inhibitory and attentional abilities on making such errors. To that end, we developed and explored a theoretical model that represents the real-time processing, cognitive processes, and execution steps involved. Differences in errors, response time, and gaze during trials that offer the potential for gain compared with no gain opportunities were measured, allowing for assessment of the cognitive processing and execution stages of the model (see Fig. 1).

1. *Perception:* Firstly, one must perceive the task and identify the correct/incorrect response, as well as the opportunity for self-serving mistakes.
2. *Cognitive processes:* Next one must process the internal considerations needed for response selection: deciding whether to act with the more automatic but sometimes less profitable task instructions or respond based on increased self-profit. If profit is chosen, cost to one’s self-image must be considered. This involves two possible conflict-management strategies:
  - Responding in a way that strategically mimics a confidant or impulsive behavior. That is, participants who have sufficiently high vigilance may engage with a fast response rate in the presence of gain, masking the fact that the opportunity for making self-serving mistakes was noticed. Specifically, we expected that reporting high attention abilities would moderate the relationship between rapid response time and self-serving mistakes.
  - Shifting gaze away from the target while acting in a non-moral way. We propose that executing this strategy requires being able to rely on proactive volitional inhibition of the need to look at the stimulus while performing a task. Specifically, we expected that reporting high inhibitory ability would moderate the relationship between gaze aversion and self-serving mistakes.
3. *Execution:* At the last step a decision is made and executed behaviorally by acting in accordance with the rule or by making a self-serving mistake.

## Method

### Participants

One hundred and thirty-three 18- to 50-year-olds (M = 25.04 ± 5.59, Female = 61%) participated in the experimental procedure in the Developmental Neuropsychology Lab. Missing demographic data were interpolated for two participants: one participant’s age was interpolated based on the group mean, and one participant’s gender was inserted based on the group mode. Data interpolation was employed to avoid listwise deletion, which has been shown to decrease the power and introduce potential bias (Roth, 1994). Participants were recruited via ads placed in community centers, universities, and on internet forums. Given the complex design of our proposed model with unique comparisons, we were unable to accurately estimate the expected effect size. The sample size was chosen based on previous studies with similar experimental procedures, which included between 30 and 90 participants. Given the complex model planned for the current analyses, we recruited a significantly larger sample of 133 participants. At the end of the *Results* section, we report a sensitivity analysis for the given sample size, showing that our sample was sufficient to detect a medium effect size.

All participants scored within the average range on the Matrices subset of the WAIS-IV intelligence test (IQ = 11.83, SD = 2.61) and answered the Levenson’s Self-Report Psychopathy Scale (Levenson et al., 1995) (mean = 1.76, SD = 0.41), to ensure intelligence and psychopathic characteristics were in the typical range. The WAIS-IV Matrices subtest shows high reliability, Cronbach’s alpha = 0.90 (Wechsler, 2008). Additionally, all participants completed the Adult ADHD Self-Report Scale (ASRS; Kessler et al., 2005). This questionnaire was developed in conjunction with the World Health Organization as a clinical screening tool meant to be used for the general population. The questionnaire includes 18 self-report questions about inattention and impulsive symptoms. Questions were designed corresponding to ADHD criteria as described in the DSM-IV. Concordance between ASRS symptom responses and clinical symptom assessment was shown to be fair to substantial for the vast majority of questions, with the majority showing an unbiased distribution of false-positives or false-negatives (ASRS; Kessler et al., 2005). The ASRS has shown high internal consistency with an overall Cronbach’s alpha = 0.95; hyperactivity/impulsivity scale Cronbach’s alpha = 0.91; and inattention subscale Cronbach’s alpha = 0.94 (Silverstein et al., 2019). Participants filled out the ASRS, and based on these responses they received continuous impulsivity (mean = 13.63, SD = 5.15), and inattention scores (mean = 14.55, SD = 5.53) representing the number of symptoms that they self-report (Adler et al., 2019).

Participants were predominantly Caucasian, characterized by average socio-economic status, and lived in central urban residences. All participants reported normal health and development and no instances of medical or psychological disorders. Before participation, all participants signed informed consent.

### Procedure

#### Adapted inhibitory control task probing cheating

An adaptation of the day-night inhibitory control task (Gerstadt et al., 1994; Ramon et al., 2011; Yarmolovsky et al., 2017) was designed to explore the cognitive mechanisms involved in decision-making through the use of a simple Stroop-like task. For the current study, the gain and no-gain congruent saliency blocks were analyzed to extract self-serving mistakes. The task was presented on a 2.66-GHz Core 2 Duo PC. All stimuli were presented using E-Prime software 2.0.10, integrated with a 23-in. Tobii TX-300 binocular eye-tracking device. The Tobii system tracks both eyes at a rated accuracy of 0.5°, sampled at 300 Hz. A successful 5-point gaze calibration was executed for each participant before beginning.

**Fig. 2:**
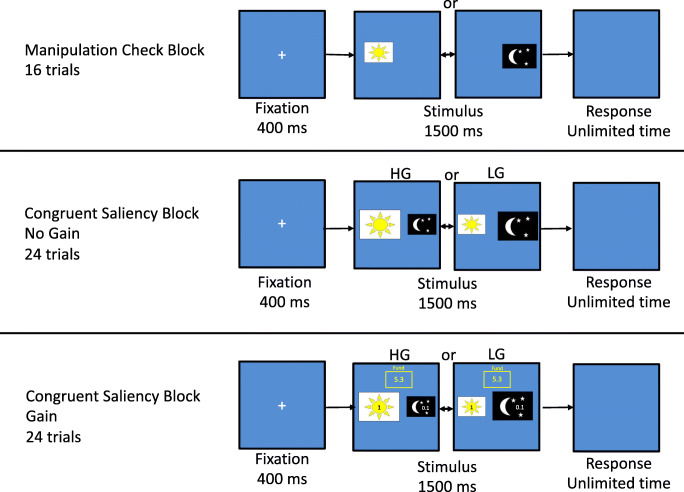
A schematic depiction of the timeline of the trial blocks adapted for probing impulsive errors as compared with self-serving mistakes. *HG* high-gain errors, *LG* low-gain errors

The modification of the day-night task incorporated selectively rewarded blocks, allowing comparisons between gain/no-gain blocks and high- and low-gain responses to differentiate between self-serving errors (dependent on gain) and impulsive errors (not dependent on gain). Participants were presented with images of either a sun or a moon on the computer screen and were instructed to choose either a sun or a moon button, via mouse click, corresponding to task instructions as quickly and as accurately as possible (see Fig. 2). First, participants underwent a manipulation-check block in which they were instructed to press a sun button when the sun appeared on the screen and a moon button when a moon appeared. This baseline block was included to ensure that participants succeeded in carrying out the basic requirements for the day-night task, including the processing of data displayed on the screen and the use of the buttons to respond. Second, participants underwent a cognitive conflict block in which they were instructed to press the button corresponding to the opposite of the image presented. The third block consisted of a congruent saliency block, for which participants were presented with a sun and a moon image of varying sizes, and they were instructed to choose the larger, more salient image.

Following were the selective gain blocks, for which participants were instructed that they would receive a reward dependent on their button presses: for each sun button press on the left key they would receive 1 NIS (equivalent to 25 c), while for each moon button press on the right key, they would receive 0.1 NIS (3 c). In these blocks, sun stimuli were labeled with 1 NIS and moon stimuli were labeled with 0.1 NIS. Additionally, cumulative earnings are displayed in a reward fund located at the top center of the monitor. Participants were informed that at the end of the experiment they would receive the sum of their total earnings. During the selective gain blocks, participants performed the same congruent saliency block procedure as described above. Additionally, under these conditions, participants underwent an incongruent saliency block, in which they were instructed to choose the smaller, less salient image, followed by a cognitive conflict block identical to the instructions in the no-gain block.

For the purpose of the current study, the manipulation checks, as well as the no-gain and gain congruent saliency blocks, were analyzed (Fig. 2). These blocks were chosen due to their slight cognitive difficulty, which provides a basic inhibitory control challenge that is still easy enough for the average adult to not take away from the cognitive effort necessary for making a self-serving mistake.

The differential reward potential offered in the current task set a platform to cheat by making profitable self-serving mistakes (i.e., errors in pressing the sun button when the moon was larger). The paradigm enabled differentiation between cheating tendencies (i.e., selective errors in response to more profitable items) and impulsive errors (errors made in response to the same stimulus in the absence of reward). During the manipulation checks, the experimenter remained in the room to ensure that the participant understood the task, while during experimental blocks, the experimenter left the room so as not to influence the participant’s ethical decision-making.

After completing the computerized tasks, participants filled out the ASRS (Kessler et al., 2005), underwent the Matrices subset of the WAIS intelligence test, and answered the Levenson’s Self-Report Psychopathy Scale measuring primary psychopathy (psychopathic emotional effect) and secondary psychopathy (psychopathic lifestyle; Levenson et al., 1995).

### Dependent variables

Two types of error rates were calculated from the computerized task: (1) Low-gain errors (LG), the percent of errors made when the participant clicked on the moon instead of the sun button, resulting in lower gain as compared with the correct response during gain opportunities; 2) High-gain errors (HG), the percent of errors made when the participant clicked on the sun instead of the moon button, resulting in increased gain as compared with the correct response during gain opportunities (Galil et al., 2019). Importantly, for the no-gain block all incorrect responses were impulsive errors, yet during the gain block LG errors represent impulsive errors only, while HG errors may include both self-serving mistakes and impulsive errors. Therefore, a comparison between HG errors in the gain and no-gain blocks enabled us to extract self-serving mistakes while taking into account impulsive errors that were not gain-driven. Self-serving mistakes were thus calculated based on the residual errors that were calculated from the regression analysis of HG errors in the no-gain block predicting HG errors in the gain block (Galil et al., 2019).

### Independent variables

The gaze-avert strategy was measured by calculating gaze differences in the gain versus the no-gain blocks. Gaze durations toward the stimulus relative to total looking time were measured both in the gain and no-gain blocks. To calculate this measure, one area of interest (AOI) was defined surrounding the stimuli presented on the screen. Given that participants rapidly move their eyes multiple times between the sun and the moon to understand which stimulus is largest and perform the cognitive task prior to executing a response (all within a mean overall response time (RT) of 842 ms.), the stimulus AOI was calculated as the total area covering the sun, the moon, and the area in between. The stimulus AOI was therefore defined as a rectangle located at the center of the screen with a width of 947.2 pixels and a height of 337.92 pixels. Gaze durations to the AOI lasting more than 20 ms were included in the analyses. Recordings of gaze durations began with stimulus presentation for each trial and ended when participants submitted a response. The dependent measure of gaze differences reflecting gaze aversion was computed using the following formula:

$$ Gaze\ Diff= Relative\ Gaze\ Duration\ Gain- Relative\ Gaze\ Duration\  No\  Gain $$

Lower gaze values indicated faster disengagement from the stimulus in the gain block compared to the no-gain block. As is common with gaze-tracking procedures (Burleson-Lesser et al., 2017), our sample included nine participants (6.7%) with missing gaze data. To limit power compromises and potential bias, data for these participants were interpolated using the group mean (Roth, 1994). Notably, findings related to gaze behaviors were significant both with and without interpolation.

For the quick RT strategy, the total RT for all responses, including both LG and HG trials, was calculated and differences were calculated between gain and no-gain blocks. RT difference was thus assessed using the following formula:

$$ RT\  Diff= Total\  RT\  Gain- Total\  RT\  No\  Gain $$

Lower RT values indicated faster response time in gain trials, independent of stimulus type, as compared with no-gain trials.

## Procedural validation

A manipulation check was run to ensure the efficacy of the congruent saliency block on this population. Paired-sample t-tests were performed to assess differences between the base-line block and the congruent saliency block. Findings showed significantly more errors in the congruent saliency no-gain block (M = 0.03, SD = 0.030) compared with the base-line block (M = 0.01, SD = 0.022), t(132) = -6.31, p < 0.00001, Cohen’s d *= -*.547). Increased rates of impulsive errors, elicited in the absence of gain in the congruent saliency block, confirmed that this block demanded higher cognitive investment.

**Fig. 3:**
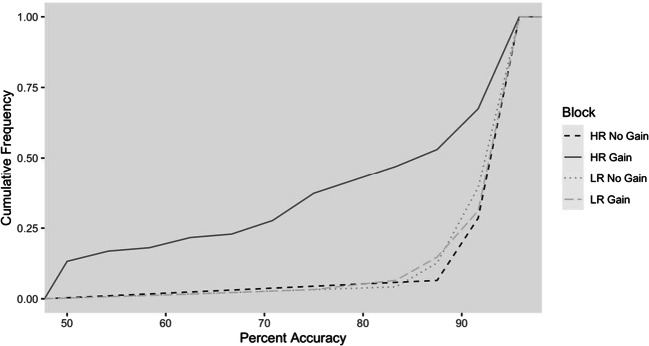
Cumulative distribution of percent accuracy as a function of block and stimulus type

Cumulative frequency rates of percent accuracy for HG and LG trials in gain and no-gain blocks are depicted in Fig. 3. The figure depicts the overall high accuracy rate due to the relatively easy cognitive task. Notably, lower levels of accuracy are only seen in the HR gain block, for which self-serving mistakes occurred.

**Fig. 4:**
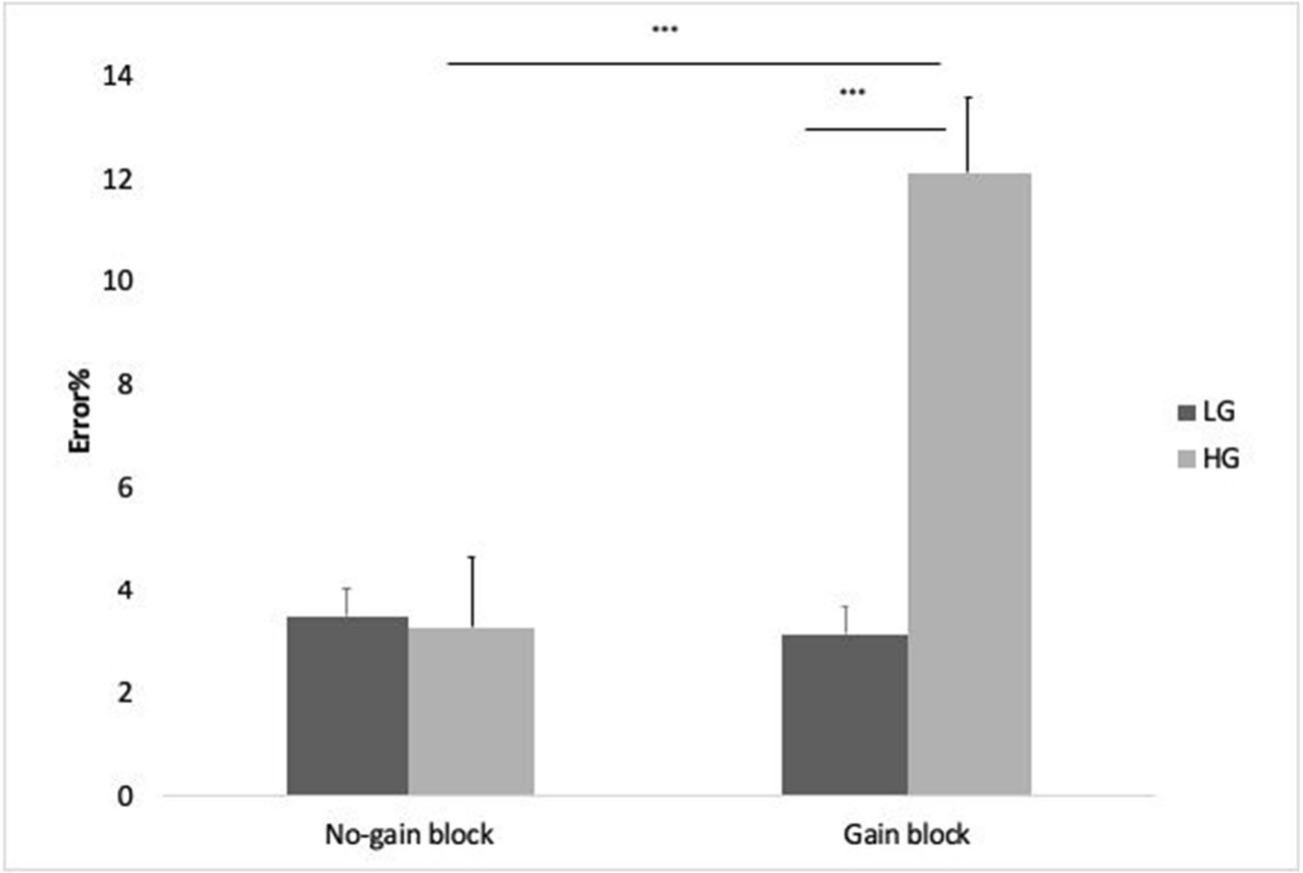
Percent errors as a function of error type and block type. Errors bars represent paired difference standard errors (Pfister & Janczyk, 2013); *** p < .001. *LG* low gain, *HG* high gain

To ensure that participants took advantage of the cheating opportunities in the task, a 2 × 2 repeated-measures analysis was conducted comparing error rate as a function of gain (gain vs. no-gain) and trial type (LG and HG). Results showed gain by error type interaction (F(1,132) = 34.7, p < 0.001, partial eta^2^ = 0.208). Post hoc comparison showed that participants made more HG errors during the gain block compared to the no-gain block (t = 8.28, p < 0.001). Additionally, more HG than LG errors were made in the gain block (t = -8.37, p < 0.001). Importantly, no error-type differences between sun and moon presses were seen in the no-gain block, suggesting that there was no bias toward one button over the other in the task (t = 0.20, p = NS; Fig. 4).

To understand whether participants pre-emptively placed their finger on the high-gain option by pressing the sun button in the gain blocks and whether learning effects were present, a 2 × 2 repeated-measures analysis was conducted comparing RT as a function of gain (gain vs. no-gain) and trial type (HG vs. LG). Findings showed an overall trial-type main effect (F(1,132) = 24.09, p < .0001, partial eta^2^ = 0.115), such that participants respond faster to the HG (left mouse press) than to the LG (right mouse press) trials, suggesting an overall bias toward the left HG mouse press. While we cannot rule out that this bias is due to pre-planned button presses to increase personal gain, we suggest that it may be more related to a bias to the more natural motor movement of left HG mouse pressing compared with right mouse key pressing (LG option). This explanation seems more likely for two reasons: (1) No accuracy differences were found between HG (left press) and LG (right press) options in the no gain blocks (see Fig. 4), suggesting that whilst it may be easier and faster to press the left rather than the right button, it does not affect the ability to respond correctly to the task in the absence of gain. (2) The gain by trial-type interaction was insignificant (F(1,132) = 1.38. p = 0.24 NA, partial eta^2^ = 0.01), suggesting that the overall bias occurred to a similar extend in both the presence and the absence of gain.

Considering potential learning effects, no significant block main effect was noted (F(1,132) = 3.85, p = 0.052 NS, partial eta^2^ = 0.03).

## Results

First, relations among the dependent variables and participants’ reported cognitive capacities were explored. Correlations and descriptive statistics between calculated variables are presented in Table 1.

**Table 1:** Means, standard deviations, and correlations

Variable	*M*	*SD*	1	2	3	4	5
1. IQ	11.83	2.61					
2. Age	25.04	5.57	-.02				
3. Inattention	14.55	5.53	-.07	-.07			
4. Hyperactivity/ Impulsivity	13.63	5.15	-.11	-.10	.67**		
5. RT Diff	-0.09	0.51	.11	-.10	.12	.08	
6. Gaze Diff	-0.04	0.14	.03	-.12	.09	.23**	.10

*M* and *SD* are used to represent mean and standard deviation, respectively. * indicates *p* < .05. ** indicates *p* < .01

Findings indicate two linear relations, the first is a moderate-strong relation between reported Hyperactivity and Inattention; the second unveils a mild link between hyperactivity/impulsivity and the gaze difference measure; leading to the final phase of the analysis of testing the full predictive model.

### Inhibitory control and Attentional as predictors of cheating strategies

A hierarchical regression model was employed with self-serving mistakes as the predicted variable (see Table 2) as a function of reported inattention and hyperactivity/impulsivity abilities and real-time gaze and button press behaviors. In the first step age and gender were entered as covariate variables to control for the variance that these measures contribute to the model. In the second step symptoms of inattention and hyperactivity/impulsivity were entered. In the third step, RT and gaze differences were entered. In the fourth step, the interactions between the two ADHD dimensions were entered to examine the effect of managing-conflict strategies on cheating. This interaction was included under the notion that the attention network is related to the executive inhibitory control network, but operates based on partially independent neuro-biological networks. Therefore, it was important for the current purposes to explore whether inattention and hyperactivity interact to predict self-serving mistakes. Finally, in the following step interactions between the two conflict-management strategies and ADHD dimensions were entered to examine how inattention and hyperactivity/impulsivity moderate the relationship between managing-conflict strategies and self-serving mistakes.

**Table 2:** Multiple regressions predicting cheating

	*Dependent variable:*			
	Self-serving mistakes			
	(1)	(2)	(3)	(4)
Age	0.0004	0.00002	-0.002	-0.003
	(-0.005, 0.006)	(-0.005, 0.005)	(-0.006, 0.002)	(-0.006, 0.001)
Gender	-0.011	-0.006	-0.015	-0.026
	(-0.069, 0.047)	(-0.064, 0.052)	(-0.058, 0.028)	(-0.067, 0.014)
Hyp/Imp		-0.008^*^	-0.002	-0.004
		(-0.015, -0.0005)	(-0.007, 0.004)	(-0.010, 0.001)
Inattention			0.003	0.001
		(-0.004, 0.010)	(-0.004, 0.006)	(-0.003, 0.007)
RT Diff			-0.042^*^	-0.104^***^
			(-0.083, -0.001)	(-0.150, -0.057)
Gaze Diff			-0.778^***^	-0.589^***^
			(-0.935, -0.621)	(-0.762, -0.415)
Hyp/Imp × Inattention				-0.0001
				(-0.001, 0.0004)
Hyp/Imp × Gaze Diff				0.043^*^
				(0.005, 0.082)
Inattention × RT Diff				0.011^***^
				(0.006, 0.017)
Inattention × Gaze Diff				-0.009
				(-0.038, 0.019)
Hyp/Imp × RT Diff				0.250
				(-0.027, 0.526)
Constant	0.000	0.000	0.000	-0.009
	(-0.028, 0.028)	(-0.027, 0.027)	(-0.020, 0.020)	(-0.030, 0.013)
R^2^ Change		0.037	0.431	0.107
F Change		2.444	51.099***	6.076***
R^2^	0.001	0.038	0.469	0.576
Residual Std. Error	0.163 (df = 130)	0.161 (df = 128)	0.121 (df = 126)	0.110 (df = 121)
F Statistic	0.095 (df = 2; 130)	1.271 (df = 4; 128)	18.543^***^ (df = 6; 126)	14.913^***^ (df = 11; 121)

^*^ p < .05; ^**^ p < .01; ^***^ p < 0.01. Values in parentheses in the upper portion of the table indicate the 95% confidence interval for each regression coefficient. Values in parentheses in the lower portion of the table represent degrees of freedom

Overall the full model was significant and explained 57.6% of the variance in self-serving mistakes *F(11,121) = 14.913, p < .001*. Notably, neither inattention nor hyperactivity/impulsivity's main effects significantly predicted cheating. Both cognitive-management strategies (quick RTs and gaze avert) did, however, significantly predict cheating. That is, both Gaze and RT differences were negatively related to cheating, explaining 26.7% (CI = 14.9–39.7) and 13.7% (CI = 4.6–26.0) of the variance, respectively. Directions suggest that increased gaze averts from the stimuli in the gain block, as well as quicker responses in the gain block as compared with the no gain block predict cheating.

Next, given the significant correlation between ADHD measures, interactions between hyperactivity/impulsivity and inattention were explored in predicting cheating. No significant interaction effect was noted between these measures and the outcome.

*Finally, we explored the moderating effects of impulsivity/hyperactivity and inattention on the relationship between conflict-management strategies and cheating behavior.*

**Fig. 5:**
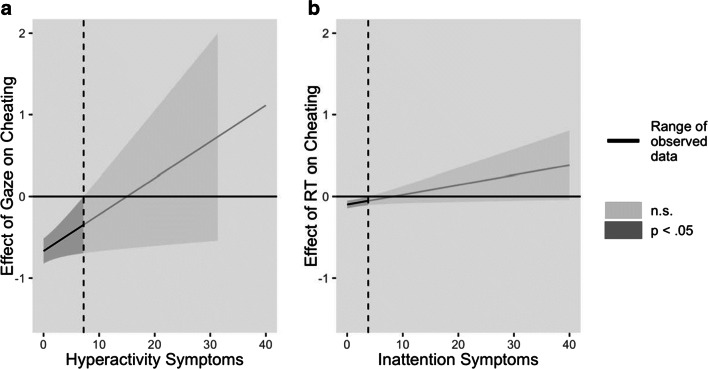
Moderation effects of hyperactivity and inattention symptoms on conflict-managing strategy behaviors

This analysis showed that the relationship between quick RT and cheating behavior was moderated by inattention. That is, when participants reported low distractibility, faster RT in the gain block compared to the no-gain block (strategy of faster RT in gain block) was significantly related to increased cheating (Fig. 5B). The interaction contributed 11.2% (CI = 3.0–23.1) of the explained variance in cheating behavior.

Further, the relationship between gaze averts and cheating behavior was moderated by hyperactivity/impulsivity. The effect was such that when participants reported low hyperactivity/impulsivity, lower gaze duration in the gain compared to the no-gain block (gaze-avert strategy during gain opportunities) was significantly related to cheating (Fig. 5A). The interaction explained an additional 3.9% (CI = 0.1–13.0) of the variance in cheating behavior.

### Sensitivity analysis

We ran a sensitivity analysis to determine the detected effect size that our sample size allowed. We used G*Power (Faul et al., 2007) to run a sensitivity test for linear multiple regression for 11 total predictors and five tested interactions. Analysis to detect 80% power and a significance level of 0.05 revealed that a sample of 133 participants was sufficient to detect a medium effect size f^2^ of 0.101 (the equivalent of R^2^ = 0.09). Therefore, the data collected supports a medium effect size similar to that seen in our current analysis.

## Discussion

The current research explored a theoretical model of real-time cheating by making self-serving mistakes, focusing on how various levels of inhibitory and attentional abilities influence responses in a cognitive-load task that offers an opportunity to make self-serving mistakes. Our findings lend support to the notion that cheating involves the reallocation of attention and inhibition resources in a controlled way, both in identifying the possibility to cheat and in using conflict-managing strategies. These strategies are of great importance in understanding the motives and barriers for deceptive behavior while focusing specifically on a fairly prevalent form of cheating – cheating by making a biased effort to succeed or self-serving mistakes.

Exploring empirically a theoretical model, the current study contributes to the literature in a number of theoretical, empirical, and applicative domains. The current study offered opportunities to make selective effort to increase gain in an effortful task, reflecting a common type of day-to-day decision-making scenario. As anticipated, most participants cheated to increase their gain, but only did so to a limited extent (Median HG errors 4.17%). These findings are consistent with studies that probed other forms of cheating (Gino et al., 2011), and extend Mazar et al.’s (2008) postulation that people act dishonestly to the degree that allows profit while still maintaining their sense of integrity, even when engaging in tasks that offer opportunities for making a selective effort to succeed.

The current data also extend the implications of findings concerning a common form of cheating previously shown in children (Galil et al., 2019) to typically functioning adults. This suggests that self-serving mistakes are expected in a wide age range from 8- to 12-year-olds (Galil et al., 2019), through 18- to 50-year-olds, speaking to the role of self-serving mistakes in human performance.

The paradigm was successful in supporting the basic assumption that participants make more profitable errors when given the opportunity in three ways: (1) low-gain errors occurred less than high-gain errors within the gain blocks; (2) high-gain errors occurred more in the gain blocks as compared to the no-gain blocks; and (3) no low-gain error differences were seen in the gain versus no-gain blocks. Notably, no differences were found between LG and HG errors in the no-gain block. Therefore, stimulus-press assignment effects did not appear to account for the effects seen.

Given this prevalent epiphenomenon, we explored specific cognitive strategies that serve to minimize the cost of making self-serving mistakes to self-image. The current study conducted an extensive exploration of the cognitive abilities involved in this form of cheating, with a focus on how inhibitory and attentional control contribute to the use of specific cognitive conflict-management strategies.

### Conflict-management strategies are supported by cognitive abilities

We explored a theoretical model that delineated the information-processing steps involved in conflict-management strategies used to make self-serving mistakes, probably without feeling bad about it. That is, after a proposed processing of the situation to understand whether or not a cheating opportunity exists, we tested how different cognitive abilities facilitate the use of conflict-management strategies. Specifically, we explored RT and gaze behavior in trials with gain opportunities as compared those with no gain. In accordance with the model, results showed that both speedy RT and gaze-avert conflict-management strategies related to cheating.

Importantly, the current research further explored the cognitive resources that enable executing these strategies, highlighting relations between reported levels of inhibitory and attentional abilities and strategic use of gaze aversion and hastened RT to effectively manage cheating-related conflicts. Both strategies of quick response and gaze aversion require both attention and inhibitory control (Schepisi et al., 2020), and both serve multiple functions; but the current theoretical model and findings from the regression model underscore that in the context of self-serving mistakes, quick response relies predominantly upon vigilant attention, while gaze avert relies on inhibitory control needed to enable disengagement from the target.

More specifically, gaze aversion predicted self-serving mistakes only for participants with high inhibitory abilities. This finding points to the cognitive mechanism underlying the gaze-shifting strategy. The phenomenon of gaze avert while cheating may be attributed to the notion that one’s dominant response is to gaze at the stimulus to succeed in a task. To intentionally avert gaze, one has to go against one’s innate response and enact high levels of inhibitory control (Boucher et al., 2007). Therefore, high impulse control is needed to suppress the urge of looking, and employ this self-serving action as a self-justification strategy, namely by limiting one’s own ability to notice the cheating-prone event. Current data show that indeed this strategy serves mostly those with high inhibitory control capacities.

Similar to the gaze-avert dependence on inhibitory control, attentional abilities moderated the relationship between RT and cheating. That is, faster RTs in the gain block as compared with no-gain trials correlated with cheating only when attentional abilities were high. This finding suggests that to successfully use the rapid RT strategy, participants must attend to multiple aspects of the trial efficiently to be able to quickly perceive the opportunity to cheat and/or decide whether or not to press the more profitable cue (Tabatabaeian et al., 2015). This requires quite a complex cognitive process: perceiving the correct response as compared with the self-serving one, considering potential gain and taking into account the risk to self-image all before making a decision at a faster rate. This supports findings by Shalvi et al. (2012) that participants need time to behave morally. Current findings suggest, for the first time, that when attention ability is high, these time-consuming considerations can occur succinctly so that participants can act quickly, imitating impulsive errors.

While previous studies have shown that people need more time to overtly lie (Foerster et al., 2019; Walczyk et al., 2003) in the current context of self-serving mistakes under a time-sensitive task that requires light cognitive load, we propose that if an individual has sufficient attentional abilities they can successfully perform the task with even quicker responses in a biased way toward personal gain. This ability allows the individual to mimic an impulsive error and aid in managing the conflict of increasing gain and preserving self-perception.

Taken together, this paper contributes to (1) the debate on whether honesty/dishonesty is the dominant response, (2) the debate on self-control and inhibition on cheating, and (3) the understudied area of cognitive justifications to maintain a positive self-concept. Underscoring the mental effort needed to cheat well, for example, to cheat at a low enough level while using strategies that cover these events, these data support the notion that acting honestly is a human dominant response, that with effort may be inhibited in people with high enough inhibitory control capacities. Secondly the study adds to the debate concerning self-regulation and inhibition on cheating by recognizing and distinguishing between impulsive errors that occur inadvertently, and self-serving mistakes that involve distributed inhibition control networks. Thirdly, the study extends the understudied area of individual differences in cognitive strategies that serve to maintain and fortify one's positive self-concept, by suggesting that self-reports of mental capacities concerning self-control, attention, and the ability to inhibit impulsive urges serve key roles in the actual execution of the cheating events. This latter finding opens the door for increasing peoples' awareness to the notion that while cheating to a moderate degree by making self-serving mistakes is prevalent in children and adults, we are endowed with different cognitive mechanisms that are not available to all in the same way. Given that the use of these cognitive mechanisms is related to an increased likelihood to cheat, educating about these effects may contribute to future development of socio-educational programs designed to reduce cheating.

### Study limitations and implications

One potential form of bias introduced in the study is the use of eye tracking. Knowing that one’s eyes are monitored may plausibly affect self-serving mistakes. While this is likely to somewhat affect performance (possibly by reducing cheating rates), the cheating blocks in the current study occur after the no-gain blocks, which may have acted as a habituation period serving to reduce the effect of the eye tracker. Manipulating the salience of the tracking device may be explored in future studies.

The use of self-report rather than direct measures of attention and inhibition calls for consideration. In the current paradigm the participant's sense of self-cognitive resources is essential. Therefore, the self-report format was probed, and revealed an important aspect of the participant's awareness and self-assessment of their ability to rely on these skills when necessary. Objective measures may produce differential results. Thus, adding such measures in future studies may enable comparisons between objective and perceived attention and inhibition abilities, providing further insight into the mechanisms involved in executing self-serving mistakes.

Finally, the fairly easy nature of the task, which was incorporated to allow participants to devote cognitive resources to make self-serving mistakes, leads to very few errors overall. Future studies with more blocks may explore trials following errors to more directly understand conflict-management responses following errors that lead to gain and those that do not.

In summary, current findings highlight two conflict-management strategies that predict self-serving mistakes: gaze aversion and quick RTs in response to gain. Further, analysis showed that attentional and inhibitory cognitive resources moderate the ability to use these strategies to execute self-serving mistakes. These findings serve to reveal the cognitive mechanisms that enable individuals to employ conflict-management strategies to execute biased responses and increase individual gain while preserving an honest self-image.

## References

[CR1] Adler LA, Faraone SV, Sarocco P, Atkins N, Khachatryan A (2019). Establishing US norms for the Adult ADHD Self-Report Scale (ASRS-v1. 1) and characterising symptom burden among adults with self-reported ADHD. International Journal of Clinical Practice.

[CR2] Babinski LM, Hartsough CS, Lambert NM (1999). Childhood conduct problems, hyperactivity-impulsivity, and inattention as predictors of adult criminal activity. The Journal of Child Psychology and Psychiatry and Allied Disciplines.

[CR3] Boucher L, Palmeri TJ, Logan GD, Schall JD (2007). Inhibitory control in mind and brain: An interactive race model of countermanding saccades. Psychological Review.

[CR4] Brown WM, Moore C (2000). Is prospective altruist-detection an evolved solution to the adaptive problem of subtle cheating in cooperative ventures? Supportive evidence using the Wason selection task. Evolution and Human Behavior.

[CR5] Bucciol A, Piovesan M (2011). Luck or cheating? A field experiment on honesty with children. Journal of Economic Psychology.

[CR6] Burleson-Lesser, K., Morone, F., DeGuzman, P., Parra, L. C., & Makse, H. A. (2017). Collective behaviour in video viewing: A thermodynamic analysis of gaze position. *PloS One*, *12*(1). 10.1371/journal.pone.0168995 PMC5207684 28045963

[CR7] Debey E, De Houwer J, Verschuere B (2014). Lying relies on the truth. Cognition.

[CR8] Ding XP, Omrin DS, Evans AD, Fu G, Chen G, Lee K (2014). Elementary school children’s cheating behavior and its cognitive correlates. Journal of Experimental Child Psychology.

[CR9] Dionisio DP, Granholm E, Hillix WA, Perrine WF (2001). Differentiation of deception using pupillary responses as an index of cognitive processing. Psychophysiology.

[CR10] Evans AD, Lee K (2011). Verbal deception from late childhood to middle adolescence and its relation to executive functioning skills. Developmental Psychology.

[CR11] Evans, A. D., & Lee, K. (2013). Lying, morality, and development. *Handbook of Moral Development*, 361-384.

[CR12] Faul F, Erdfelder E, Lang A-G, Buchner A (2007). G* Power 3: A flexible statistical power analysis program for the social, behavioral, and biomedical sciences. Behavior Research Methods.

[CR13] Fletcher J, Wolfe B (2009). Long-term consequences of childhood ADHD on criminal activities. The Journal of Mental Health Policy and Economics.

[CR14] Foerster A, Wirth R, Berghoefer FL, Kunde W, Pfister R (2019). Capacity limitations of dishonesty. Journal of Experimental Psychology: General.

[CR15] Fu G, Heyman GD, Qian M, Guo T, Lee K (2016). Young children with a positive reputation to maintain are less likely to cheat. Developmental Science.

[CR16] Galil A, Yarmolovsky J, Gidron M, Geva R (2019). Cheating behavior in children: Integrating gaze allocation and social awareness. Journal of Experimental Child Psychology.

[CR17] Gerstadt CL, Hong YJ, Diamond A (1994). The relationship between cognition and action: performance of children 312–7 years old on a stroop-like day-night test. Cognition.

[CR18] Gino F, Schweitzer ME, Mead NL, Ariely D (2011). Unable to resist temptation: How self-control depletion promotes unethical behavior. Organizational Behavior and Human Decision Processes.

[CR19] Halevy N, Chou EY (2014). How decisions happen: Focal points and blind spots in interdependent decision making. Journal of Personality and Social Psychology.

[CR20] Hilbig BE, Thielmann I (2017). Does everyone have a price? On the role of payoff magnitude for ethical decision making. Cognition.

[CR21] Hinshaw SP, Simmel C, Heller TL (1995). Multimethod assessment of covert antisocial behavior in children: Laboratory observations, adult ratings, and child self-report. Psychological Assessment.

[CR22] Isaacowitz DM (2006). Motivated gaze: The view from the gazer. Current Directions in Psychological Science.

[CR23] Kessler RC, Adler L, Ames M, Demler O, Faraone S, Hiripi E, Howes MJ, Jin R, Secnik K, Spencer T (2005). The World Health Organization Adult ADHD Self-Report Scale (ASRS): a short screening scale for use in the general population. Psychological Medicine.

[CR24] Kochanska G, Murray K, Coy KC (1997). Inhibitory control as a contributor to conscience in childhood: From toddler to early school age. Child Development.

[CR25] Kotaman H (2017). Impact of rewarding and parenting styles on young children’s cheating behavior. European Journal of Developmental Psychology.

[CR26] Leib M, Pittarello A, Gordon-Hecker T, Shalvi S, Roskes M (2019). Loss framing increases self-serving mistakes (but does not alter attention). Journal of Experimental Social Psychology.

[CR27] Levenson MR, Kiehl KA, Fitzpatrick CM (1995). Assessing psychopathic attributes in a noninstitutionalized population. Journal of Personality and Social Psychology.

[CR28] Mazar N, Amir O, Ariely D (2008). The dishonesty of honest people: A theory of self-concept maintenance. Journal of Marketing Research.

[CR29] Paulhus DL, Dubois PJ (2015). The link between cognitive ability and scholastic cheating: A meta-analysis. Review of General Psychology.

[CR30] Pfister R, Janczyk M (2013). Confidence intervals for two sample means: Calculation, interpretation, and a few simple rules. Advances in Cognitive Psychology.

[CR31] Pfister R, Wirth R, Schwarz KA, Steinhauser M, Kunde W (2016). Burdens of non-conformity: Motor execution reveals cognitive conflict during deliberate rule violations. Cognition.

[CR32] Pfister R, Wirth R, Weller L, Foerster A, Schwarz KA (2019). Taking shortcuts: Cognitive conflict during motivated rule-breaking. Journal of Economic Psychology.

[CR33] Pittarello A, Leib M, Gordon-Hecker T, Shalvi S (2015). Justifications shape ethical blind spots. Psychological Science.

[CR34] Pittarello A, Motro D, Rubaltelli E, Pluchino P (2016). The relationship between attention allocation and cheating. Psychonomic Bulletin & Review.

[CR35] Ramon D, Geva R, Goldstein A (2011). Trait and state negative affect interactions moderate inhibitory control performance in emotionally loaded conditions. Personality and Individual Differences.

[CR36] Roth PL (1994). Missing data: A conceptual review for applied psychologists. Personnel Psychology.

[CR37] Schepisi M, Porciello G, Aglioti SM, Panasiti MS (2020). Oculomotor behavior tracks the effect of ideological priming on deception. Scientific reports.

[CR38] Schweitzer ME, Hsee CK (2002). Stretching the truth: Elastic justification and motivated communication of uncertain information. Journal of Risk and Uncertainty.

[CR39] Shalvi S, Dana J, Handgraaf MJ, De Dreu CK (2011). Justified ethicality: Observing desired counterfactuals modifies ethical perceptions and behavior. Organizational Behavior and Human Decision Processes.

[CR40] Shalvi S, Eldar O, Bereby-Meyer Y (2012). Honesty requires time (and lack of justifications). Psychological Science.

[CR41] Sibley MH, Pelham WE, Molina BS, Gnagy EM, Waschbusch DA, Biswas A, MacLean MG, Babinski DE, Karch KM (2011). The delinquency outcomes of boys with ADHD with and without comorbidity. Journal of Abnormal Child Psychology.

[CR42] Sibley MH, Pelham WE, Molina BS, Waschbusch DA, Gnagy EM, Babinski DE, Biswas A (2010). Inconsistent self-report of delinquency by adolescents and young adults with ADHD. Journal of Abnormal Child Psychology.

[CR43] Silverstein MJ, Faraone SV, Alperin S, Leon TL, Biederman J, Spencer TJ, Adler LA (2019). Validation of the expanded versions of the adult ADHD self-report scale v1. 1 symptom checklist and the adult ADHD investigator symptom rating scale. Journal of attention disorders.

[CR44] Speights D, Hilinski M (2005). Return fraud and abuse: How to protect profits. Retailing Issues Letter.

[CR45] Tabatabaeian M, Dale R, Duran ND (2015). Self-serving dishonest decisions can show facilitated cognitive dynamics. Cognitive Processing.

[CR46] Talwar V, Gordon HM, Lee K (2007). Lying in the elementary school years: Verbal deception and its relation to second-order belief understanding. Developmental Psychology.

[CR47] Talwar V, Lee K (2008). Social and cognitive correlates of children’s lying behavior. Child Development.

[CR48] Walczyk JJ, Roper KS, Seemann E, Humphrey AM (2003). Cognitive mechanisms underlying lying to questions: Response time as a cue to deception. Applied Cognitive Psychology: The Official Journal of the Society for Applied Research in Memory and Cognition.

[CR49] Wechsler, D. (2008). *WAIS-IV technical and interpretive manual*. Pearson.

[CR50] Wells JT (1999). A fistful of dollars. Security Management.

[CR51] Wise SL, Kong X (2005). Response time effort: A new measure of examinee motivation in computer-based tests. Applied Measurement in Education.

[CR52] Yarmolovsky J, Szwarc T, Schwartz M, Tirosh E, Geva R (2017). Hot executive control and response to a stimulant in a double-blind randomized trial in children with ADHD. European Archives of Psychiatry and Clinical Neuroscience.

[CR53] Zhao L, Heyman GD, Chen L, Lee K (2017). Praising young children for being smart promotes cheating. Psychological Science.

